# Atypical monkeypox presentation in a previously vaccinated MSM HIV-positive adult

**DOI:** 10.1007/s15010-022-01967-9

**Published:** 2023-01-13

**Authors:** Verena Crosato, Melania Degli Antoni, Ilaria Izzo, Carlo Cerini, Francesca Pennati, Maurizio Gulletta, Silvia Odolini, Lina Rachele Tomasoni, Alberto Matteelli, Francesco Castelli

**Affiliations:** 1 Unit of Infectious and Tropical Diseases, Department of Clinical and Experimental Sciences, University of Brescia, Brescia, Italy; 2 Unit of Infectious and Tropical Diseases, ASST Spedali Civili, Brescia, Brescia, Italy

**Keywords:** Monkeypox, HIV, MSM, Vaccine, Misdiagnosis

## Abstract

The outbreak of monkeypox virus (MPXV) in non-endemic countries is an international public health emergency, and the diversity in manifestations poses challenges for early diagnosis and isolation. We describe an atypical case of monkeypox (MPX) in a 46-year-old homosexual male living with HIV. He reported 1-day duration fever, a lesion on his chin that, over a period of 18 days, had gradually enlarged and ulcerated. Biopsy examination performed at an external centre revealed pyoderma gangrenosum, unconfirmed at a subsequent biopsy. When he reported to our hospital outpatients’ clinic the chin lesion had a diameter of 5 × 5 cm, necrotic margins and ulcerated base and signs of superinfection. He was admitted for further investigations. Three swabs collected from pharynx, skin and chin lesion resulted positive for MPXV. He had a favourable clinical course and was discharged soon after. Pending the achievement of optimal vaccination coverage in at-risk groups, early identification and isolation of infectious patients represent the cornerstones of the containment strategy. Atypical cases of MPX manifestations are not uncommon, particularly in patients with HIV infection. A high level of suspicion should be maintained to identify infectious cases at an early stage and avoid further spread of the infection.

## Introduction

Prior to 2022, monkeypox (MPX) human cases were rarely reported outside the continent of Africa [1]. A local outbreak of 47 cases was reported in the USA in 2003 following the importation of small mammals from Ghana [2]. The first multi-country outbreak in non-endemic countries is currently underway, and it represents an international public health emergency [3]. The current outbreak is exposing gaps in our knowledge of monkeypox, and clinicians should be aware of atypical manifestations of the disease and the risk of misdiagnosis.

In this case report, we describe the atypical manifestation of monkeypox in a previously incompletely vaccinated HIV-positive patient, who also became the first confirmed case of MPX at our clinic at Spedali Civili General Hospital in Brescia.

## Case description

We describe a 46-year-old homosexual male living with HIV, followed at our clinic since 2000 with a nadir of T-CD4^+^ count of 103 cells/µl at the time of diagnosis. He was on antiretroviral therapy with dolutegravir/lamivudine, with undetectable HIV-RNA levels and a T-CD4^+^ count persistently greater than 500 cells/µl. In his clinical history, there were no AIDS-defining events, but he presented with multiple episodes of syphilis (last treatment in 2016), including a diagnosis of neurosyphilis that was adequately treated in 2008. He was incompletely vaccinated against smallpox during childhood (he reported a single dose of vaccine).

Five days after attending a concert in a disco pub in Milan in early July 2022, he reported a single febrile episode (> 38 °C), with spontaneous resolution. He denied occasional sexual intercourses during that time period.

About 5 days after the febrile episode and around 7–10 days after the weekend in Milan, he noticed a vesicular lesion on the left side of his chin; in the following 2 weeks, this first lesion was followed by a diffuse papulo-vesicular rash which involved his trunk, limbs, genitalia, oral cavity, palms and soles.

**Fig. 1:**
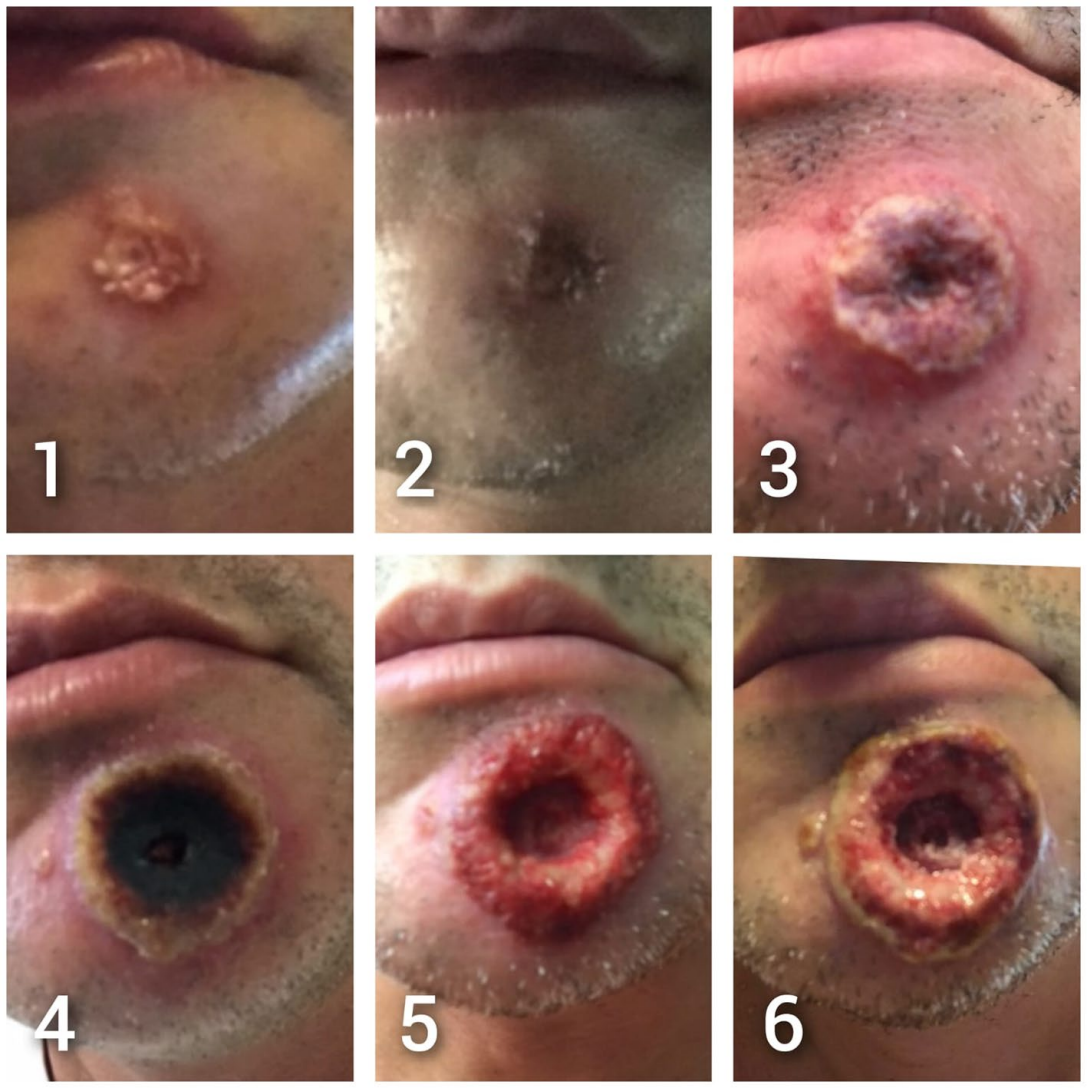
Overtime evolution of the MPX chin lesion as documented photographically by the patient himself during the 18 days since it first appeared. **1.** Day 1: initial ulceration of 1.5 × 2 mm with apical keratinization. **2.** Day 3: central erosion with darkening of the borders, size unchanged. **3.** Day 9: expansion of the lesion’s size and signs of superinfection with appearance of satellite lesions. Size 3 × 3 cm. **4.** Day 13: formation of necrotic layer with persistence of central umbilication. The lesion started deepening and enlarged further to a diameter of 3.5 × 3.5 mm. **5.** Day 15: surgical removal of the necrotic layer, showing worsening of the lesion, which is now reddish and secretive. Size 4.5 × 4.5 mm. **6.** Day 18: the margins start to necrotize and the patient consulted to our outpatient clinic

Due to constant itching, the patient reported scratching the chin lesion intensely for some days, until it worsened and gradually enlarged, ulcerated, and was covered by a necrotic layer (Fig. 1).

Ten days after the first appearance of the lesion, he consulted a maxillofacial surgeon who prescribed an X-ray of the jaw (which did not show bone involvement) and performed a biopsy of the lesion, with a histological diagnosis of pyoderma gangrenosum.

In the following days, worried about the further growth of the chin lesion, the patient also accessed the emergency room, where the necrotic component was removed, and he was told to apply self-medications. He was prescribed antibiotics (amoxicillin/clavulanate) and antivirals (acyclovir) and discharged.

Frightened by the lack of improvement over the following days, the patient presented to our HIV outpatient clinic for a consultation. The chin lesion was about 5 × 5 cm in diameter, with necrotic margins and an ulcerated base, with a depth of at least 1 cm (Fig. 1).

On medical examination, the rest of his body was covered by papule–pustular lesions at different stages of development, mostly with initial apical crusting. Despite the patient denied exposure to unprotected sex, given the high suspicion of MPX infection, he was admitted to our Infectious Diseases ward for the diagnostic process. He was isolated in a single room according to our internal monkeypox protocols.

On admission to the ward, the patient underwent nasopharyngeal swab for antigen detection of SARS-CoV-2 (resulted negative), a chin lesion swab for germs (resulted negative) and serology for syphilis (resulted positive Treponema pallidum hemagglutinin (TPHA) with rapid plasma reagin (RPR) test 1:4; unchanged since March 2021). On suspicion of bacterial superinfection of the chin lesion, empirical antibiotic therapy with ampicillin/sulbactam and clindamycin was initiated, with gradual regression of the necrosis and initial healing of the base.

A new biopsy of the chin lesion was performed for histological and culture tests. Bacterial culture of the chin lesion was negative, and the histological examination showed unspecific signs of inflammation in an advanced and healing stage. The PCR for MPXV from pharyngeal, skin and chin lesions swabs resulted positive 2 days after admission (18 days after the appearance of the first lesion on the chin).

Given his overall good clinical conditions, the progressive healing of the skin lesions and the possibility of self-isolation at home, the patient was discharged 4 days after admission and continued the follow-up at our dedicated outpatient clinic. One week after discharge, the lesion on his chin was visibly healing and shrinking in size; the rest of his body was mostly covered with crusts, though a few new papules were still observed, mainly on his trunk. The mandatory isolation was lifted 3 weeks after the initial consultation at our clinic, upon evidence that all crusts had fallen off and there was no evidence of new cutaneous lesions. Three weeks after the onset of the first symptoms, the partner, who had close contacts and lived in the same household, remained asymptomatic.

## Discussion

MPX is an emergent infectious disease caused by a virus belonging to the same family as the smallpox virus. It causes a disease that resembles human smallpox but is mostly characterized by milder symptoms and a much lower fatality rate [4]. It mostly affects people living in sub-Saharan Africa, and just a few cases were reported in the past in travellers from endemic regions [5]. It spreads through close contact with skin lesions of affected individuals [3, 6, 7].

In May 2022, a large outbreak of human monkeypox disease was reported worldwide, with over 50.000 confirmed cases in 99 countries throughout the world. Some fatal cases were also reported, especially in young men [7].

Awaiting vaccination coverage of at-risk categories, given the contagiousness of infected patients whose skin lesions release live virions, early identification and isolation of contagious patients is of pivotal importance to stop the outbreak [8]. In our case, the patient developed the disease 18 days before it was properly diagnosed and, in that period, he attended his workplace, the emergency room and public recreation places with his partner and other friends. It is not yet known whether he might have infected others with MPX. For this reason, MPX needs to be kept in mind as an emerging infectious disease and included in the differential diagnosis of suspicious skin lesions, particularly in patients who report having had risk factors for viral transmission.

Atypical cases of MPX are not rare in the literature. In some cases, both the absence of prodromal symptoms and the presence of herald skin lesions only at the point of sexual contact were observed [9–11]. This suggests that human-to-human transmission through close physical contact in sexual networks plays a key role in the current outbreak. Moreover, asynchronous skin lesions manifestations are described [9, 10]. An international collaboration across 16 countries reported 528 infections that were diagnosed between 27 April and 24 June 2022. In the study, 95% of these patients presented with a rash, 73% had anogenital lesions, and 41% had mucosal lesions. A tenth of the patients (54) had only a single genital lesion. Another unexpected finding was that 15% had anal or rectal pain (or both). Accordingly, the current international case definition needs to be expanded including these atypical presentations to reduce misdiagnoses [12, 13].

The fact that the main lesion was the one on the chin is likely the result of manipulation by the patient. The spread to the whole body of vesicular–papular exanthem without sparing hands and feet strongly argued in favour of MPX. We hypothesized that the initial lesion was the one on the chin and that the others arising on the rest of the body resulted from viral spreading over the first 18 days of disease.

Although the diagnosis of pyoderma gangrenosum was not confirmed by the second chin biopsy, we cannot exclude a MPXV-triggered immune dysfunction leading to such an atypical evolution of the main lesion, considering the coinfection with HIV.

There are limited data about the MPX/HIV coinfection. Previous studies in Africa stated that people with untreated HIV infection had more extensive and longer-lasting lesions, more complications and an overall worse outcome [14]. Up to date, there are only few case reports or case series, but a recent analysis suggested that coinfected people may present with a pool of atypical manifestations, for instance, whitish papules in a kissing lesion configuration in the perianal area [15]. The largest study of confirmed MPX cases to date found that HIV infection was not linked to monkeypox severity [12].

Most of the reported cases in the current outbreak are men who have sex with men (MSM), many of whom are also infected by HIV. The fact that the population at risk of the two infections is largely overlapping, can justify why many cases of MPX have been reported in the HIV-positive population.

According to CDC, the smallpox vaccine is deemed to confer effective protection (85%), but these data are mainly based on individuals with recent exposure to monkeypox and vaccinated using a third-generation vaccine [7]. Our patient reported having been vaccinated with a dose of smallpox in his childhood. It is possible that HIV-related immunosuppression at the time of T-CD4^+^ count nadir be responsible for a lack of protective antibody response against monkeypox despite prior immunization. Further studies are needed to prove the efficacy of previous vaccination and whether this can be abolished by HIV-related immunosuppression.

## Data Availability

All the original data presented in the study are included in the manuscript.
